# From Millimeters to Micrometers; Re-introducing Myocytes in Models of Cardiac Electrophysiology

**DOI:** 10.3389/fphys.2021.763584

**Published:** 2021-10-27

**Authors:** Karoline Horgmo Jæger, Andrew G. Edwards, Wayne R. Giles, Aslak Tveito

**Affiliations:** ^1^Simula Research Laboratory, Lysaker, Norway; ^2^Department of Physiology and Pharmacology, Faculty of Medicine, University of Calgary, Calgary, AB, Canada

**Keywords:** computational modeling, computational electrophysiology, cardiac modeling, action potential propagation, cell based model, EMI model, cardiac disease, conduction abnormalities

## Abstract

Computational modeling has contributed significantly to present understanding of cardiac electrophysiology including cardiac conduction, excitation-contraction coupling, and the effects and side-effects of drugs. However, the accuracy of *in silico* analysis of electrochemical wave dynamics in cardiac tissue is limited by the homogenization procedure (spatial averaging) intrinsic to standard continuum models of conduction. Averaged models cannot resolve the intricate dynamics in the vicinity of individual cardiomyocytes simply because the myocytes are not present in these models. Here we demonstrate how recently developed mathematical models based on representing every myocyte can significantly increase the accuracy, and thus the utility of modeling electrophysiological function and dysfunction in collections of coupled cardiomyocytes. The present gold standard of numerical simulation for cardiac electrophysiology is based on the bidomain model. In the bidomain model, the extracellular (E) space, the cell membrane (M) and the intracellular (I) space are all assumed to be present everywhere in the tissue. Consequently, it is impossible to study biophysical processes taking place close to individual myocytes. The bidomain model represents the tissue by averaging over several hundred myocytes and this inherently limits the accuracy of the model. In our alternative approach both E, M, and I are represented in the model which is therefore referred to as the EMI model. The EMI model approach allows for detailed analysis of the biophysical processes going on in functionally important spaces very close to individual myocytes, although at the cost of significantly increased CPU-requirements.

## 1. Introduction

In this brief Perspective we present our approach for cell-based modeling of the cardiac syncytium. We point out the limitations of the standard, averaged models, and then illustrate how our approach can be used to reveal fundamental dynamics of a range of important, but incompletely understood mechanisms of arrhythmia. The main advantage of the cell-based approach is significantly increased accuracy, and the main disadvantage is significantly increased cost of the computations, and increased cost of the associated software development.

## 2. A Quantitative Understanding of Most Arrhythmias Remains Elusive

Optimal functioning of the heart relies on distinct electrochemical waves repetitively traversing the entire four chambered structure. Even transient perturbations to this wave, referred to as arrhythmias, can be life-threating and have therefore been subject to intense research efforts for at least a century. In general, both atrial and ventricular arrhythmias are well-defined clinical phenomena, but for many, precise explanations of their origin and/or maintenance—how the coordinated activity of myocytes becomes pathologically discoordinated—is lacking. This significantly compromises clinical efforts to restore normal cardiac conduction (Spach et al., 2007; Nattel and Dobrev, 2017; Heijman et al., 2021).

## 3. The Limit of Accuracy of the Standard Models of Cardiac Electrophysiology

The adult human heart contains several billion myocytes (Tirziu et al., 2010). Spatial averaging (homogenization) has been an invaluable tool for creating computationally tractable mathematical models of electrochemical wave dynamics including the action potential (AP) and details of change in intracellular calcium (Ca^2+^). This has led to broad adoption of two standard models of cardiac electrophysiology; the monodomain and bidomain models. Since the late 1970's, the bidomain model (see e.g., Tung, 1978; Neu and Krassowska, 1993; Franzone et al., 2014) has been the gold standard for simulating electrochemical conduction in cardiac tissue, and has often been approximated by the slightly simpler monodomain model (Sundnes et al., 2007; Clayton and Panfilov, 2008; Vigmond et al., 2008). Coupled with models of myocyte membrane ion transport, these models have been very successful in accounting key aspects of the electrical activity in cardiac tissue. Furthermore, owing to dramatically increased computing power and substantially improved numerical methods, it is now possible to solve the bidomain model to convergence. A spatial resolution of Δ*x* ≈ 0.25 mm is generally considered to be sufficient to compute the solution of the model (Xie et al., 2004; Clayton and Panfilov, 2008; Niederer S. A. et al., 2011; Niederer S. et al., 2011). This mesh resolution represents tissue blocks containing ~980 myocytes each (assuming myocyte volume of 16 pL, including associated extracellular space, see numerical example below; Nygren et al., 1998). Since the converged solution can be computed, further mesh refinement does not provide greater insight to the physiological processes or dynamic characteristics of normal conduction. However, electrochemical conduction disturbances that underlie arrhythmia are not well-approximated by normal planar wave conduction. Rotor wave dynamics provide one important and ubiquitous example in which key details of the wave dynamics in smaller tissue regions are likely to be important. In this paradigm, the trajectory of a rotating wave is determined by a phase singularity and an adjacent region of maximum wavefront curvature. The singularity “meanders” around a central core and the maximum curvature dictates that meander in a manner that determines measurable clinical properties of the arrhythmia (e.g., dominant frequency of fibrillation). Importantly, even in large mammal (sheep) myocardium, rotor wave cores measured to be as small as 3 mm^2^ (Mandapati et al., 2000) are sufficient to support arrhythmic activity. The rotor wave core is one case in which understanding sub-millimeter-scale dynamics is likely to be important for defining and managing macroscopic outcomes. In addition, other dynamical conditions (e.g., ectopic focus), and heterogeneous innervation or non-uniform drug effects also likely rely on dynamics that are not adequately captured by models constructed to replicate only planar wave dynamics.

As mentioned above, the bidomain model has been very successful in simulating propagation of electrochemical waves in cardiac tissue on the macroscale when the transmembrane conductances are assumed to vary only on that scale. However, key properties of gap junctions can vary individually. Therefore, averaging can give misleading results. In order to illustrate this, let us consider a case where a long, one-dimensional strand in which myocytes are connected via gap junctions. With normal gap junctions and a strong electrical stimulation at one end of the strand, an excitation wave will be initiated and move along all myocytes both for EMI and for the bidomain models. Also, if the parameters of the models are properly adjusted, the conduction velocity of this wave will be similar. Suppose next that one gap junction is significantly disturbed leading to almost infinite resistance from one myocyte to the next. At that point the wave will stop when using the EMI model since the impaired junction will cause a complete conduction block. For the bidomain model however, change of one gap junction connection will mean little when the average is computed and the wave will only move a little slower, but not stop. Although this is a specific single example and the bidomain model can probably be adjusted (e.g., by mesh alignment) to compute the correct solution, it does illustrate the fundamental difficulties of averaging.

## 4. Cell-Based Models of Electrophysiology

**Figure 1:**
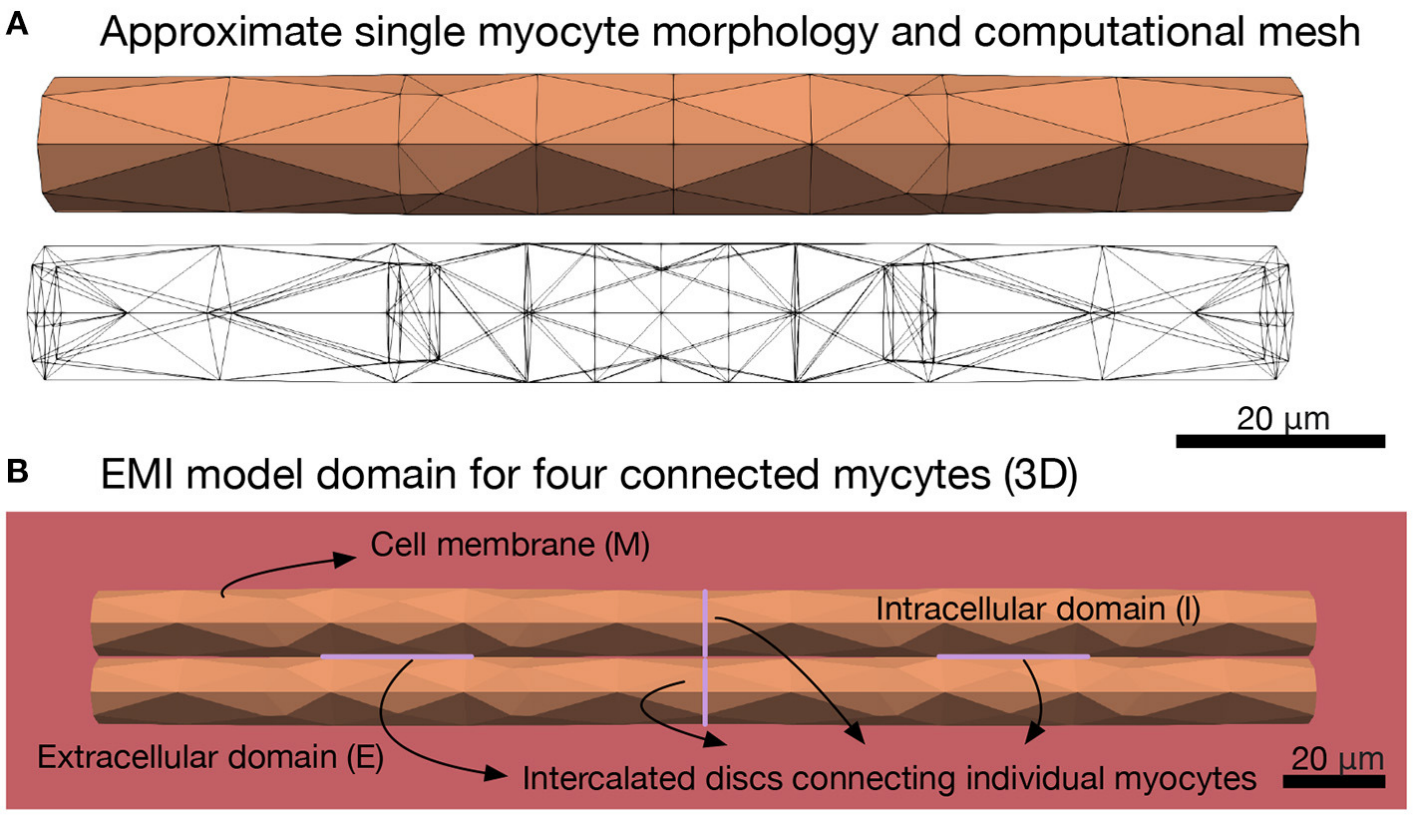
Illustration of the EMI model domain. **(A)** Shows the approximate cylindrical geometry and associated mesh of a single myocyte of length 120 μm and diameter ranging from 13 to 14 μm (Nygren et al., 1998). **(B)** Shows an illustration of the different components of the EMI model domain for an example collection of four connected myocytes. The domain consists of a number of myocytes surrounded by an extracellular space. The cell membrane is defined at the interface between the intracellular and extracellular spaces and intercalated discs with gap junctions are defined at the interface between adjacent myocytes. All computations presented here are in 3D.

A second major assumption of the monodomain and bidomain models is that the extracellular space, the myocyte membrane and the intracellular space are all present everywhere in the cardiac tissue. This represents a very significant simplification since individual myocytes can be ignored, but it also imposes strict limits on the range of problems than can be realistically studied using these models. For example it excludes the ability to understand how spatially localized ion channel expression may impact macroscopic conduction, and this is known to be fundamental to the contribution of sodium channel activation to cardiac conduction (Rivaud et al., 2017; Jæger et al., 2019). In short, classical cardiac models of electrochemical conduction are relevant for phenomena at scales from several millimeters to centimeters, and under conditions approximating planar conduction; whereas dynamics occurring among groups of myocytes at or below the millimeter scale are likely to be key to many mechanisms of arrhythmia. Here, we further develop our approach described in Tveito et al. (2017a), Jæger et al. (2019), and Jæger and Tveito (2021) and show that it is feasible to devise mathematical models at the micrometer level and thereby include every individual myocyte in the *in silico* tissue. We refer to this as the EMI model since it explicitly represents the extracellular (E) space, the cell membrane (M), and the intracellular (I) space. Figure 1B illustrates the different components of the spatial EMI model domain. Our results demonstrate the utility of the EMI model by addressing key aspects of how arrhythmias can arise in cardiac tissue. Specifically, we show that re-entry can arise in very small, partially de-coupled, collections of myocytes.

## 5. Cell-Based Models Can Resolve Micro-Reentry in a Simulated Pulmonary Vein Sleeve

**Figure 2:**
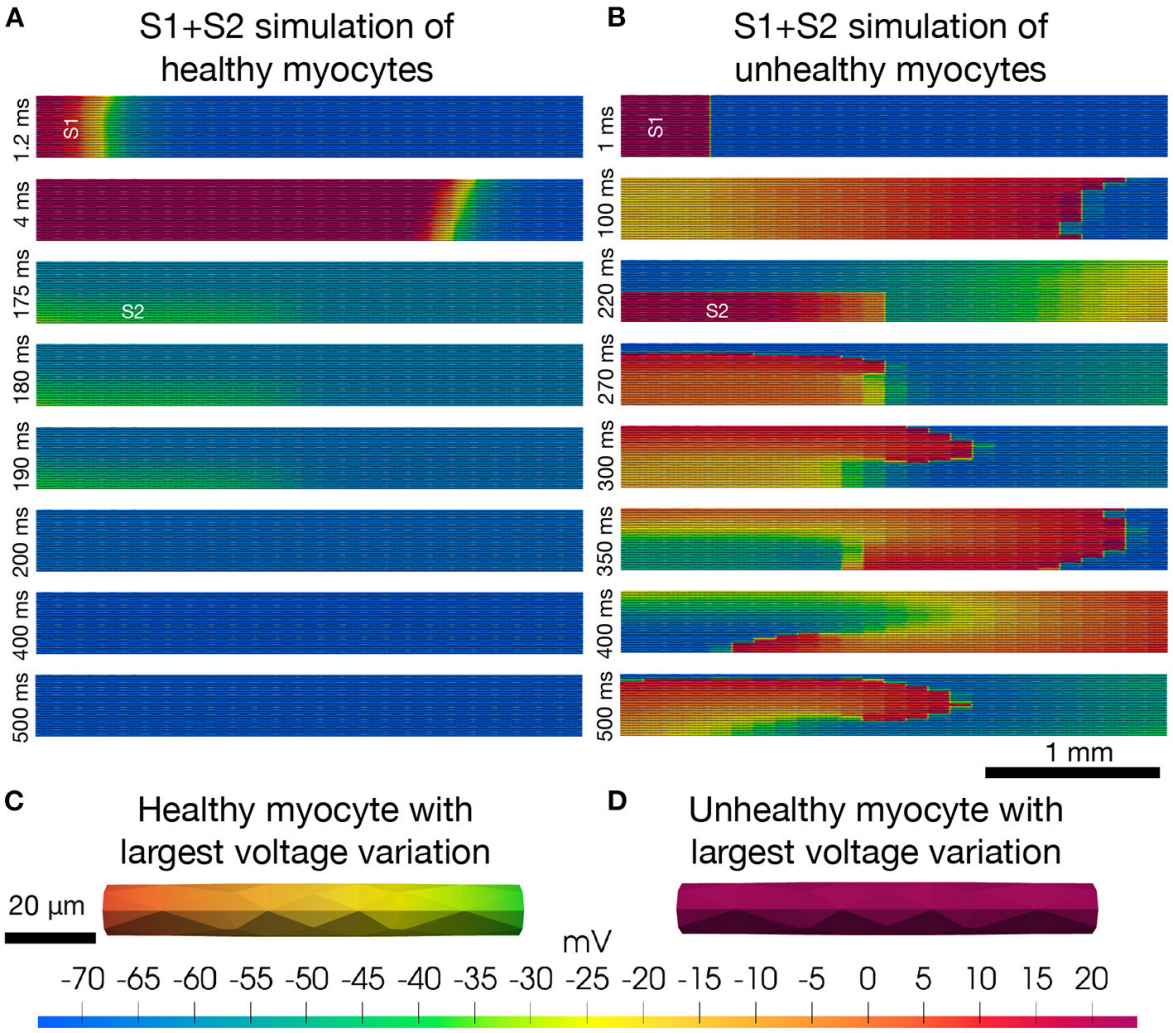
Re-entry in a cell-based model. The upper panels **(A,B)** show the results (intracellular potential) of S1+S2 stimulations (see, e.g., Spach et al., 2007) of a collection of 25×25 myocytes. The simulations are started by stimulation of the four leftmost rows of cells (marked by S1) and a resulting depolarization wave propagates through the tissue from left to right. After the center cell is repolarized to −60 mV, the lower left quarter of cells (marked by S2) are stimulated, potentially initiating a second depolarization wave through the tissue. In the healthy case **(A)**, the S2 stimulation does not produce a second wave, whereas in the unhealthy case **(B)**, the solution evolves into to a stable re-entrant wave that continues indefinitely. In the lower panels (**C,D)**, we show the solution of the single myocyte with the largest observed variation in intracellular potential during the simulation at the time point when the largest variation is observed. The maximum single cell variation is much larger in the healthy case **(C)** than in the unhealthy case **(D)**.

In atrial fibrillation, the arrhythmia often begins in the “sleeve” of the pulmonary veins of the left atrium (see e.g., Haissaguerre et al., 1998). In particular, the pulmonary vein/left atrial “junction” is assumed to be a driver of atrial fibrillation partially due to the highly heterogeneous intercellular coupling in this region (Koura et al., 2002; Pfenniger, 2020), and regions of structural conduction discontinuities (Hamabe et al., 2003). Heterogeneities in small collections of myocytes represented by cell-to-cell variations in membrane ion currents and intercellular coupling can initiate arrhythmias. Arrhythmias caused by heterogeneities can clearly not be accurately represented by models where the heterogeneities are accounted for only by averages. In Figure 2, we illustrate a collection of 25 × 25 myocytes covering an area of 1 mm^2^ with associated volume of 18 nL. We simulate the electrochemical wave (AP and Ca^2+^-transient) in this heterogeneous syncytium using the EMI model, combined with a myocyte membrane model representing canine left pulmonary vein myocytes. To represent phenotypic heterogeneity in the tissue, the maximum conductance of each ionic current are in fact given as a random combination of these values in models of the left atrial free wall myocyte and the pure pulmonary vein myocyte. These differences are derived from detailed studies of the two myocyte populations (Ehrlich et al., 2003; Melnyk et al., 2005). Furthermore, to replicate one known property of diseased atrial tissue the intercellular resistance is increased (reducing gap-junction conductance) again chosen randomly for each myocyte-to-myocyte coupling in the right panel of Figure 2, similarly to Cherry et al. (2007). The simulations reveal that stable re-entrant waves can develop and propagate in very small collections of unhealthy myocytes under conditions of markedly reduced intercellular coupling. Further details of the model and method of solution are provided in Supplementary Material.

## 6. EMI Represents a Grand Challenge in High Performance Computing

In the finite element mesh used in the simulations reported here, the average edge length of a computational element is about 10 μm. This means that the average mesh block has a volume of 1 pL. This should be compared to a mesh block for the bidomain model which is (0.25mm)^3^ = 15, 600 pL, and to the volume of a myocyte which in the present model is 16 pL. Because of the significantly increased resolution, the EMI model is only applicable to relatively small collections of myocytes in a syncytium. Using the finite element code described in the Supplementary Material, we find that the computing times needed for simulating one time step of size 0.001 ms with 100, 625, or 2,500 myocytes are 19, 125, and 568 ms, respectively. Accordingly, the computing time requirements per myocyte are 0.19, 0.20, and 0.23 ms, respectively. These computing efforts fulfills the requirements given by Feynman (2018) who stated that the cost of a computation should be proportional to the space-time volume of the problem under consideration. For the EMI model, this means that the computational efforts should be proportional to the number of myocytes included in our simulations. This optimality criterion is met by the present finite element code and also by a finite difference method applied earlier (Tveito et al., 2017a; Jæger et al., 2021; Kuchta et al., 2021). However, these computing times remain prohibitive for many interesting and important applications, and further work on optimizing computing methods and application of larger HPC-facilities is definitely needed. Important steps toward improved solution technology is underway in the EU-funded *Microcard project (www.microcard.eu)*.

Since the computing efforts needed to solve the EMI model is proportional to the number of myocytes in the simulations, it is obvious that there are well defined restrictions on the classes of application can be studied by this model. Restricted collections of myocytes like the special nodes of the cardiac conduction system (SA- and AV-nodes, Purkinje fibers etc.), areas like the outlet of the pulmonary veins, border zones associated with ischemic regions can be analyzed. In contrast, whole organ simulations are probably only accessible for very small model animals, such as the zebrafish and perhaps atrial or right ventricular tissue from adult mice.

## 7. Still Averaging

Finally, it should be pointed out that even with explicit representation of all myocytes in a simulation, the EMI model is still based on averaging of the processes taking place at a length scale that remains unreachable for tissue scale simulations. Specifically, Ca^2+^-driven arrhythmia is known to involve criticality in the ensemble behavior of subcellular (micron scale) Ca^2+^-handling structures. Detailed models of sub-cellular Ca^2+^-dynamics have been developed (see, e.g., Colman et al., 2020) but application of these models in syncytium of myocytes remains challenging.

## 8. Discussion

At present, almost all tissue simulations of cardiac electrophysiology are based on models like the bidomain and monodomain models which entail spatial averaging and related limitations in spatial resolution. However, these simulations used to be performed using models where individual myocytes were coupled in cables and the electrical conduction was assumed to be one dimensional (see, e.g., Lieberman et al., 1973; Joyner, 1982; Rudy and Quan, 1987; Quan and Rudy, 1990; Shaw and Rudy, 1997). These models based on representation of individual myocytes have also been extended to two and three space dimensions (Roberts et al., 2008; Stinstra et al., 2010; Hubbard and Henriquez, 2012) while retaining the myocytes in the model. However, these models are complex from an implementational point of view, and also quite demanding in terms of CPU requirements. Our EMI model is based on formulations suggested by Krassowska and Neu (1994), Henríquez et al. (2013), and Agudelo-Toro and Neef (2013) and we first applied it to evaluate the accuracy of classical models of the transmembrane potential of neurons (see Tveito et al., 2017b).

As explained above, a major disadvantage of the EMI approach compared to the bidomain or monodoman model is the CPU requirements needed to perform simulations. It is, however, worthwhile to recall that this also used to be a disadvantage of the “averaged” models. In 1984, it was estimated (see Barr and Plonsey, 1984) that simulating 10 ms using 10^6^ mesh points, applying a brute force method, would take 3,000 years! But already in 2006, a 26 ×10^6^ nodes simulation (600 ms) was performed in only 2 days (Potse et al., 2006), and a few years later even more challenging computations were performed in 5 min (see Niederer S. A. et al., 2011). Today, such simulations are performed routinely. A comprehensive and interesting discussion of the history of tissue models is provided in Henriquez (2014).

We suggest that a series of important physiological questions can be addressed using the EMI model. In particular, we believe the EMI model can yield important new insights into mechanism of action of cardiac drugs, how early after depolarizations (EADs) and delayed after depolarizations (DADs) are generated in collections of myocytes, how deterioration of gap-junction coupling can modulate cardiac conduction, how reentry can be generated in very small collections of myocytes, and whether ephaptic coupling can maintain conduction in diseased tissue. Furthermore, we believe that a more complete understanding of the role of T-tubules in ventricular myocytes can be achieved if the EMI model is applied along with improved spatial modeling of ionic concentration changes in the associated restricted extracellular spaces (see Ellingsrud et al., 2020; Setterberg et al., 2021).

## Data Availability Statement

The original contributions presented in the study are included in the article/Supplementary Material, further inquiries can be directed to the corresponding author/s.

## Author Contributions

KJ and AT did the mathematical modeling, performed the numerical simulations, and wrote the paper. AE and WG reviewed and edited the paper. All authors contributed to the article and approved the submitted version.

## Conflict of Interest

The authors declare that the research was conducted in the absence of any commercial or financial relationships that could be construed as a potential conflict of interest.

## Publisher's Note

All claims expressed in this article are solely those of the authors and do not necessarily represent those of their affiliated organizations, or those of the publisher, the editors and the reviewers. Any product that may be evaluated in this article, or claim that may be made by its manufacturer, is not guaranteed or endorsed by the publisher.
