# Supplementary material for: From Millimeters to Micrometers; Re-introducing Myocytes in Models of Cardiac Electrophysiology
Source: Front Physiol. 2021 Oct 27;12:763584. doi: 10.3389/fphys.2021.763584 (PMC8578869; doi:10.3389/fphys.2021.763584)
Supplement: Supplementary file 3 [file Data_Sheet_1.PDF]

S1 Supplementary Information

Contents

S1 Supplementary Information 1

S1.1 Formulation of the EMI model 1

S1.1.1 Nonuniform distribution of ion channels 2

S1.1.2 Cell-to-cell variations in ion channel densities 3

S1.1.3 Representation of unhealthy cells 3

S1.1.4 S1+S2 stimulation protocol 4

S1.1.5 Numerical solution of the EMI model 4

S1.2 Formulation of the base model for the membrane dynamics . 6

S1.2.1 Membrane currents 6

S1.2.2 Ca^{2+} dynamics 10

S1.2.3 Na^{+} dynamics 12

S1.2.4 Nernst equilibrium potentials 12

S1.1 Formulation of the EMI model

The EMI model used in our simulations is a cell-based mathematical model that represents the electric potential in a domain consisting of a number of cells (Ω_i^k), surrounded by an extracellular space (Ω_e). The cell membrane (Γ_k) is defined as the boundary between Ω_i^k and Ω_e, and the boundary between two neighboring cells (Ω_i^k and $\Omega_i^{\tilde{k}}$) defines an intercalated disc ($\Gamma_{k,\tilde{k}}$). See Figure S1 for an illustration of the domain for two connected exemplary cells. The EMI model takes the form

$$
\begin{aligned}
\nabla \cdot \sigma_i \nabla u_i^k &= 0 \quad \text{in } \Omega_i^k, & n_e \cdot \sigma_e \nabla u_e &= -n_i^k \cdot \sigma_i \nabla u_i^k \equiv I_m^k && \text{at } \Gamma_k, \\
\nabla \cdot \sigma_e \nabla u_e &= 0 \quad \text{in } \Omega_e, & v_t^k &= \tfrac{1}{C_m} I_m^k - I_{\text{ion}}^k && \text{at } \Gamma_k, \\
u_e &= 0 \quad \text{at } \partial\Omega_e, & u_i^k - u_i^{\tilde{k}} &= w^k && \text{at } \Gamma_{k,\tilde{k}}, \\
u_i^k - u_e &= v^k \quad \text{at } \Gamma_k, & n_i^{\tilde{k}} \cdot \sigma_i \nabla u_i^{\tilde{k}} &= -n_i^k \cdot \sigma_i \nabla u_i^k \equiv I_{k,\tilde{k}} && \text{at } \Gamma_{k,\tilde{k}}, \\
s_t^k &= F^k \quad \text{at } \Gamma_k & w_t^k &= \tfrac{1}{C_g}(I_{k,\tilde{k}} - I_{\text{gap}}^k) && \text{at } \Gamma_{k,\tilde{k}},
\end{aligned}
\tag{1}
$$

for all cells k and neighboring cells $\tilde{k}$ [1]. Here, u_e (in mV) is the electric potential in Ω_e, and u_i^k (in mV) is the electric potential in Ω_i^k. Moreover, v^k (in mV) is the membrane potential of cell k, defined at Γ_k, and w^k (in mV) is the potential difference between cell k and its neighboring cell $\tilde{k}$, defined at $\Gamma_{k,\tilde{k}}$. Furthermore, σ_i and σ_e (in mS/cm) are the conductivity of

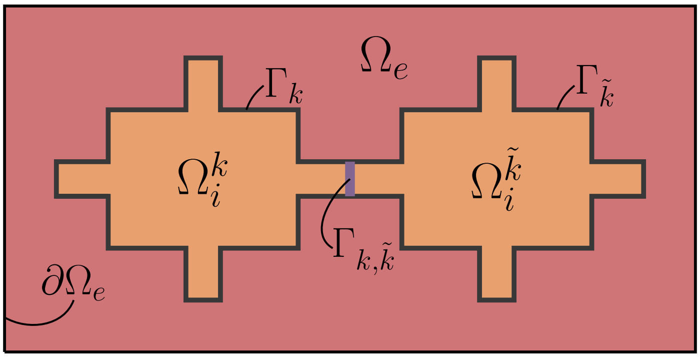

Figure S1: Two-dimensional illustration of an EMI model domain with a caricature cell, Ω_i^k, connected to a neighbouring cell, $\Omega_i^{\tilde{k}}$, by an intercalated disc, $\Gamma_{k,\tilde{k}}$. The cells are surrounded by an extracellular space, Ω_e, and the interface between the cells and the extracellular space defines the cell membranes, Γ_k and $\Gamma_{\tilde{k}}$, for Ω_i^k and $\Omega_i^{\tilde{k}}$, respectively. Note that in our simulations, the domain is three-dimensional.

the extracellular and intracellular spaces, respectively, and C_m and C_g (in μF/cm^2) are the specific capacitance of the cell membrane and the intercalated discs, respectively. In addition, I_{ion}^k (in μA/μF) represents the sum of ionic current densities through ion channels, pumps and exchangers in the cell membrane. The model for these currents involves a set of additional state variables, referred to as s^k, and the dynamics of these additional variables are governed by F^k. The models used for I_{ion}^k and F^k are described in more detail in Section S1.2. Similarly, I_{gap}^k (in μA/cm^2) represents the current density through gap junctions in the intercalated discs between cells. These currents are given by the simple passive model

$$I_{\text{gap}}^k = \frac{1}{R_{\text{gap}}} w^k, \tag{2}$$

where R_{gap} (in kΩcm^2) is the resistance of the gap junctions. The values used for the EMI model parameters are specified in Table S1.

S1.1.1 Non uniform distribution of ion channels of the membrane of each cell

The ion channels responsible for the I_{Na} current have been shown to be nonuniformly distributed on the membrane of cardiomyocytes. Especially,

Parameter	Value
C_m	1 μF/cm^2
C_g	0.5 μF/cm^2
σ_i	4 mS/cm
σ_e	20 mS/cm
R^*_{gap} (healthy)	0.0005 kΩcm^2
R^*_{gap} (unhealthy)	0.5–1.5 kΩcm^2
Δt	0.001 ms

Table S1: Parameter values used in the EMI model simulations.

these channels have been reported to preferentially localize close to the cell ends [2, 3, 4, 5, 6, 7]. In our simulations, 50% of the I_{Na} channels are located close to the cell ends in the longitudinal direction, as indicated in [8, 9, 10]. More specifically, the conductance value of the I_{Na} current is set to be spatially dependent, following the approach of [11, 12].

S1.1.2 Cell-to-cell variations in ion channel densities

In the simulations of the main paper, we consider a collection of cardiomyocytes located at the junction between the pulmonary vein and the left atrium. The density of certain ion channels have been shown to differ between pulmonary vein and left atrial cardiomyocytes (see [13] and Table S6), and we assume that the properties of the cardiomyocytes in the pulmonary vein-left atrial junction is a mix of the properties of the pulmonary vein and left atrial cardiomyocytes. For each cell in the collection, we draw a random number $a \in [0, 1]$ from a uniform distribution, and let the ion channel conductances be given by

$$g = a \cdot g_{\text{PV}} + (1 - a) \cdot g_{\text{LA}}, \tag{3}$$

where g_{PV} and g_{LA} refer to the pulmonary vein and left atrial version of the conductance values, respectively. The conductance values assumed to be different between pulmonary vein and left atrial cardiomyocytes are specified in Table S6.

S1.1.3 Representation of unhealthy cells — random distribution of gap junction channels

In the right panel of Figure 2 of the main text, we consider a case of unhealthy cells based on assumed impaired cell-to-cell connections. This is represented

by increasing the value of the gap junction resistance (see Table S1). In addition, the cell-to-cell connections are assumed to be non uniform, in the sense that the gap junction resistance vary between the individual intercalated discs. For each intercalated disc, we draw a random number $b \in [0, 1]$ from a uniform distribution, and let the gap junction resistance be given by

$$R_g = b \cdot R_g^{\max} + (1 - b) \cdot R_g^{\min}, \tag{4}$$

where $R_g^{\max} = 1.5$ kΩcm^2 and $R_g^{\min} = 0.5$ kΩcm^2.

S1.1.4 S1+S2 stimulation protocol

The EMI model simulations investigating the inducibility of rotor waves are performed using an S1+S2 stimulation protocol, as described in [14, 15]. Specifically, we first stimulate the four leftmost columns of cells and let a propagating wave travel across the domain in the direction from left to right. Next, when the membrane potential in a cell in the center of the domain reaches a membrane potential below −60 mV, we stimulate the bottom left quarter of the domain, and observe whether a rotor wave is induced.

S1.1.5 Numerical solution of the EMI model

In order to solve the system of equations (1) numerically, we apply the operator splitting algorithm introduced in [16, 17]. This algorithm split the coupled system (1) into standard elliptic problems that can be solved separately for the extracellular domain and for each cell. Thus, well-developed solution methods for such standard problems can be applied. The steps of the splitting algorithm is summarized in Algorithm 1. We use one inner and outer iteration ($N_{\text{it}} = M_{\text{it}} = 1$). For more details on the splitting algorithm, see [16].

We solve the EMI model equations using the finite element method. More specifically, the solver is implemented using the MFEM finite element library [18, 19] with linear elements. The ODE systems (S1) for the membrane nodes are solved using a first-order generalized Rush-Larsen scheme [20, 21]. The code for this scheme is generated using the Gotran code generator [22]. The linear systems arising from the finite element discretization of the systems (S2) and (S3) are solved using the conjugate gradient method with a symmetric Gauss-Seidel preconditioner (see, e.g., [23]). The ODE equations (S1) for each membrane node and the linear system (S2) for each cell are solved in parallell using OpenMP parallelization [24].

Initial conditions: $v^{k,0}$, $s^{k,0}$, $w^{k,0}$, u_e^0 for all k.

for $n = 1, \dots, N_t$ **:**

Step 1: For all k, find $s^{k,n}$ and $\bar{v}^k$ at the nodes of the membrane Γ_k of cell k by solving a time step Δt from $(s^{k,n-1}, v^{k,n-1})$ of

$$\begin{aligned} v_t^k &= -I_{\text{ion}}(v^k, s^k), \\ s_t^k &= F(v^k, s^k). \end{aligned} \tag{S1}$$

Define $\bar{u}_e = u_e^{n-1}$, $\bar{w}^k = w^{k,n-1}$.

for $j = 1, \dots, N_{\text{it}}$ **:**

Step 2:

for $m = 1, \dots, M_{\text{it}}$ **:**

For every k, find $\bar{u}_i^k$ by solving

$$\begin{aligned} \nabla \cdot \sigma_i \nabla \bar{u}_i^k &= 0 && \text{in } \Omega_i^k, \\ \bar{u}_i^k + \tfrac{\Delta t}{C_m} n_i^k \cdot \sigma_i \nabla \bar{u}_i^k &= \bar{v}^k + \bar{u}_e && \text{at } \Gamma_k, \\ -n_i^k \cdot \sigma_i \nabla \bar{u}_i^k &= \tfrac{1}{R_g} \bar{w}^k + C_g \tfrac{\bar{w}^k - w^{k,n-1}}{\Delta t} && \text{at } \Gamma_{k,\tilde{k}}, \end{aligned} \tag{S2}$$

where $\tilde{k}$ denotes each of the neighboring cells of cell k.

Update $\bar{w}^k = \bar{u}_i^k - \bar{u}_i^{\tilde{k}}$ at $\Gamma_{k,\tilde{k}}$ for all k and $\tilde{k}$.

end

Step 3: Find $\bar{u}_e$ by solving

$$\begin{aligned} \nabla \cdot \sigma_e \nabla \bar{u}_e &= 0 && \text{in } \Omega_e, \\ \bar{u}_e &= 0 && \text{at } \partial\Omega_e^D, \\ n_e \cdot \sigma_e \nabla \bar{u}_e &= 0 && \text{at } \partial\Omega_e^N, \\ n_e \cdot \sigma_e \nabla \bar{u}_e &= -n_i^k \cdot \sigma_i \nabla \bar{u}_i^k && \text{at } \Gamma_k \text{ for all } k. \end{aligned} \tag{S3}$$

end

Define $u_e^n = \bar{u}_e$, $u_i^{k,n} = \bar{u}_i^k$, $w^k = \bar{w}^k$ for all k.

Step 4: Define $v^{k,n} = u_i^{k,n} - u_e^n$ at Γ_k for all k.

end

Algorithm 1: Operator splitting algorithm for the EMI model from [16].

S1.2 Formulation of the base model for the membrane dynamics

In this section, we describe the formulation of the base model for the membrane dynamics. In this formulation, the membrane potential (v) is given in units of mV, as above, and the Ca^{2+} and Na^+ concentrations are given in units of mM. All currents are given in units of μA/μF, and the ionic fluxes are expressed as mmol/ms per total cell volume (i.e., in units of mM/ms). The parameters of the model are all given in Tables S2–S9. In particular, the adjustment factors used to scale the model from the default canine left atrial version to the pulmonary vein version are found in Table S6.

This base model formulation was adapted from the ones in [25, 26]. The main difference is that three currents, I_{Kur}, I_{KACh} and I_{bNa}, are included and the conductances of the currents and fluxes are adjusted to represent atrial, as apposed to ventricular, cardiomyocytes.

S1.2.1 Membrane currents

In our base model formulation, the membrane currents are given by

$$\begin{aligned} I_{\text{ion}} =& I_{\text{Na}} + I_{\text{NaL}} + I_{\text{CaL}} + I_{\text{to}} + I_{\text{Kr}} + I_{\text{Ks}} + I_{\text{K1}} + I_{\text{NaCa}} + I_{\text{NaK}} \\ &+ I_{\text{pCa}} + I_{\text{bCa}} + I_{\text{bCl}} + I_{\text{bNa}} + I_{\text{f}} + I_{\text{Kur}} + I_{\text{KACh}} + I_{\text{stim}}, \end{aligned} \tag{5}$$

where I_{stim} is an applied stimulus current given as a constant current of size -40 A/F applied for some selected cells at some selected points in time (see Section S1.1.4) until the membrane potential reaches a value of -10 mV.

In general, the currents through the voltage-gated ion channels on the cell membrane are given on the form

$$I = go(v - E),$$

where g is the channel conductance, v is the membrane potential and E is the equilibrium potential of the channel. Moreover, o is the open probability of the channels, which is given on the form $o = \prod_i z_i$, where z_i are gating variables. These gating variables are either given as an explicit function of the membrane potential or governed by equations of the form

$$z_i' = \frac{1}{\tau_{z_i}}(z_{i,\infty} - z_i). \tag{6}$$

The parameters τ_{z_i} and $z_{i,\infty}$ are specified for each of the gating variables of the model in Table S10.

Fast sodium current The formulation of the fast sodium current is based on the model formulation given in [27]. The current is given by

$$I_{\mathrm{Na}} = g_{\mathrm{Na}} o_{\mathrm{Na}} (v - E_{\mathrm{Na}}), \tag{7}$$

where the open probability is given by

$$o_{\mathrm{Na}} = m^3 j, \tag{8}$$

and m and j are gating variables governed by equations of the form (6).

Late sodium current The formulation of the late sodium current, I_{NaL}, is based on [28] and is given by

$$I_{\mathrm{NaL}} = g_{\mathrm{NaL}} o_{\mathrm{NaL}} (v - E_{\mathrm{Na}}), \tag{9}$$

where the open probability is given by

$$o_{\mathrm{NaL}} = m_L h_L, \tag{10}$$

and m_L and h_L are gating variables governed by equations of the form (6).

Transient outward potassium current The formulation of the transient outward potassium current, I_{to}, is based on [29] and is given by

$$I_{\mathrm{to}} = g_{\mathrm{to}} o_{\mathrm{to}} (v - E_{\mathrm{to}}), \tag{11}$$

where the open probability is given by

$$o_{\mathrm{to}} = q_{\mathrm{to}} r_{\mathrm{to}}, \tag{12}$$

and q_{to} and r_{to} are gating variables governed by equations of the form (6).

Rapidly activating potassium current The formulation of the rapidly activating potassium current, I_{Kr}, is based on [29] and is given by

$$I_{\mathrm{Kr}} = g_{\mathrm{Kr}} o_{\mathrm{Kr}} (v - E_{\mathrm{K}}), \tag{13}$$

where

$$o_{\mathrm{Kr}} = x_{\mathrm{Kr1}} x_{\mathrm{Kr2}}, \tag{14}$$

and the dynamics of x_{Kr1} and x_{Kr2} are governed by equations of the form (6).

Slowly activating potassium current The formulation of the slowly activating potassium current, I_{Ks}, is based on [27] and is given by

$$I_{\mathrm{Ks}} = g_{\mathrm{Ks}} o_{\mathrm{Ks}} (v - E_{\mathrm{Ks}}), \tag{15}$$

where

$$o_{\mathrm{Ks}} = x_{\mathrm{Ks}}^2, \tag{16}$$

and the dynamics of x_{Ks} is governed by an equation of the form (6).

Inward rectifier potassium current The formulation of the inward rectifier potassium current, I_{K1}, is based on [30, 31], and is given by

$$I_{\mathrm{K1}} = g_{\mathrm{K1}} \left(\frac{0.059764(v - E_{\mathrm{K}} + 0.582854)}{0.754829 + e^{0.0767156(v - E_{\mathrm{K}} + 0.582854)}} - 0.0114237 \right). \tag{17}$$

Ultrarapid delayed rectifier potassium current The formulation of the ultrarapid delayed rectifier potassium current, I_{Kur}, is based on [32, 33] and is given by

$$I_{\mathrm{Kur}} = g_{\mathrm{Kur}} f_{\mathrm{Kur}} o_{\mathrm{Kur}} (v - E_{\mathrm{K}}), \tag{18}$$

where f_{Kur} is given by

$$f_{\mathrm{Kur}} = 0.005 + \frac{0.05}{1 + e^{-\frac{v-15}{13}}} \tag{19}$$

and

$$o_{\mathrm{Kur}} = x_{\mathrm{Kur1}}^3 \cdot x_{\mathrm{Kur2}}. \tag{20}$$

The dynamics of x_{Kur1} and x_{Kur2} are governed by equations of the form (6).

Time-dependent hyperpolarization-activated potassium current The base model includes a formulation (from [34]) of the time-dependent hyperpolarization-activated potassium current found in dog. As in [34], we refer to the current as I_{KACh} because it has been suggested to be similar to the acetylcholine-activated current. The current is given by

$$I_{\mathrm{KACh}} = g_{\mathrm{KACh}} f_{\mathrm{KACh}} o_{\mathrm{KACh}} (v - E_{\mathrm{K}}), \tag{21}$$

where

$$f_{\mathrm{KACh}} = \frac{1}{0.1 + e^{0.078(V+19.97)}}, \tag{22}$$

and the dynamics of $o_{\mathrm{KACh}} = x_{\mathrm{KACh}}$ is governed by an equation of the form (6).

Hyperpolarization activated funny current The formulation for the hyperpolarization activated funny current, I_f, is based on [29] and is given by

$$I_\text{f} = g_\text{f} o_\text{f} (v - E_\text{f}), \tag{23}$$

where

$$o_\text{f} = x_\text{f}, \tag{24}$$

and the dynamics of x_f is governed by an equation of the form (6).

L-type Ca^{2+} current The formualtion for the L-type Ca^{2+} current, I_CaL, is based on the formulation in [27] and is given by

$$I_\text{CaL} = g_\text{CaL} \left(Q_{10}^\text{CaL}\right)^{Q_p} o_\text{CaL} \frac{(2F)^2 v}{RT} \frac{0.341 c_d e^{\frac{2Fv}{RT}} - 0.341 c_e}{e^{\frac{2Fv}{RT}} - 1}, \tag{25}$$

where

$$o_\text{CaL} = df(1 - f_\text{Ca}), \tag{26}$$

and the dynamics of d, f and f_Ca are governed by equations of the form (6).

Background currents The formulation of the background currents, I_bCa, I_bNa and I_bCl, are based on [27] and are given by

$$I_\text{bCa} = g_\text{bCa}(v - E_\text{Ca}), \tag{27}$$

$$I_\text{bNa} = g_\text{bNa}(v - E_\text{Na}), \tag{28}$$

$$I_\text{bCl} = g_\text{bCl}(v - E_\text{Cl}). \tag{29}$$

Sodium-calcium exchanger The formulation of the Na$^+$-Ca^{2+} exchanger current, I_NaCa, is based on [27] and is given by

$$I_\text{NaCa} = \bar{I}_\text{NaCa} \left(Q_{10}^\text{NaCa}\right)^{Q_p} \frac{e^{\frac{\nu F v}{RT}} [\text{Na}^+]_i^3 c_e - e^{\frac{(\nu-1)Fv}{RT}} [\text{Na}^+]_e^3 c_{sl}}{s_\text{NaCa} \left(1 + \left(\frac{K_\text{act}}{c_{sl}}\right)^2\right) \left(1 + k_\text{sat} e^{\frac{(\nu-1)Fv}{RT}}\right)}, \tag{30}$$

where

$$s_\text{NaCa} = K_{\text{Ca},i} [\text{Na}^+]_e^3 \left(1 + \left(\frac{[\text{Na}^+]_i}{K_{\text{Na},i}}\right)^3\right) + K_{\text{Na},e}^3 c_{sl} \left(1 + \frac{c_{sl}}{K_{\text{Ca},i}}\right) + K_{\text{Ca},e} [\text{Na}^+]_i^3 + [\text{Na}^+]_i^3 c_e + [\text{Na}^+]_e^3 c_{sl}.$$

Sarcolemmal Ca^{2+} pump The formulation of the current through the sarcolemmal Ca^{2+} pump, I_{pCa}, is based on [27] and is given by

$$I_{\mathrm{pCa}} = \bar{I}_{\mathrm{pCa}} \left(Q_{10}^{\mathrm{pCa}}\right)^{Q_p} \frac{c_{sl}^2}{K_{\mathrm{pCa}}^2 + c_{sl}^2}. \tag{31}$$

Sodium-potassium pump The current through the Na^+-K^+ pump, I_{NaK}, is based on [27] and is given by

$$I_{\mathrm{NaK}} = \bar{I}_{\mathrm{NaK}} \left(Q_{10}^{\mathrm{NaK}}\right)^{Q_p} \frac{f_{\mathrm{NaK}}}{1 + \left(\frac{K_{\mathrm{Na,i}}^{\mathrm{NaK}}}{[\mathrm{Na}^+]_{\mathrm{i}}}\right)^4} \frac{[\mathrm{K}^+]_{\mathrm{e}}}{[\mathrm{K}^+]_{\mathrm{e}} + K_{\mathrm{K,e}}}, \tag{32}$$

where

$$f_{\mathrm{NaK}} = \frac{1}{1 + 0.12e^{-0.1\frac{Fv}{RT}}} + \frac{0.037}{7}\left(e^{\frac{[\mathrm{Na}^+]_{\mathrm{e}}}{67}} - 1\right) e^{-\frac{Fv}{RT}}. \tag{33}$$

S1.2.2 Ca^{2+} dynamics

The intracellular Ca^{2+} dynamics are governed by the model introduced in [25]

$$\frac{dc_d}{dt} = \frac{1}{V_d}(J_{\mathrm{CaL}} - J_d^b - J_d^c), \qquad \frac{db_d}{dt} = \frac{1}{V_d} J_d^b, \tag{34}$$

$$\frac{dc_{sl}}{dt} = \frac{1}{V_{sl}}(J_e^{sl} - J_{sl}^c - J_{sl}^b + J_s^{sl}), \qquad \frac{db_{sl}}{dt} = \frac{1}{V_{sl}} J_{sl}^b, \tag{35}$$

$$\frac{dc_c}{dt} = \frac{1}{V_c}(J_{sl}^c + J_d^c - J_c^n - J_c^b), \qquad \frac{db_c}{dt} = \frac{1}{V_c} J_c^b, \tag{36}$$

$$\frac{dc_s}{dt} = \frac{1}{V_s}(J_n^s - J_s^{sl} - J_s^b), \qquad \frac{db_s}{dt} = \frac{1}{V_s} J_s^b, \tag{37}$$

$$\frac{dc_n}{dt} = \frac{1}{V_n}(J_c^n - J_n^s). \tag{38}$$

Here, c_d is the concentration of free Ca^{2+} in the dyad, b_d is the concentration of Ca^{2+} bound to a buffer in the dyad, c_{sl} is the concentration of free Ca^{2+} in the sub-sarcolemmal (SL) compartment, b_{sl} is the concentration of Ca^{2+} bound to a buffer in the SL compartment, c_c is the concentration of free Ca^{2+} in the bulk cytosol, b_c is the concentration of Ca^{2+} bound to a buffer in the bulk cytosol, c_s is the concentration of free Ca^{2+} in the junctional sarcoplasmic reticulum (jSR), b_s is the concentration of Ca^{2+} bound to a buffer in the jSR, and c_n is the concentration of free Ca^{2+} in the network sarcoplasmic reticulum (nSR). The expressions for the fluxes are specified below.

Flux through the SERCA pumps The flux from the bulk cytosol to the nSR through the SERCA pumps is based on [27] and given by

$$J_c^n = \bar{J}_{\text{SERCA}} \left(Q_{10}^{\text{SERCA}}\right)^{Q_p} \frac{\left(\frac{c_c}{K_c}\right)^2 - \left(\frac{c_n}{K_n}\right)^2}{1 + \left(\frac{c_c}{K_c}\right)^2 + \left(\frac{c_n}{K_n}\right)^2}. \tag{39}$$

Flux through the RyRs The flux from the jSR to the SL compartment is given by

$$J_s^{sl} = J_{\text{RyR}} + J_{\text{leak}}, \tag{40}$$

where J_{RyR} is the flux through the active RyR channels and J_{leak} is the flux through passive RyR channels that are always open, given by

$$J_{\text{RyR}} = p \cdot r \cdot \alpha_{\text{RyR}}(c_s - c_{sl}), \tag{41}$$

$$J_{\text{leak}} = \gamma_{\text{RyR}} \cdot \alpha_{\text{RyR}}(c_s - c_{sl}), \tag{42}$$

respectively. Here, p represents the open probability of the active RyR channels and is given by

$$p = \frac{c_d^3}{c_d^3 + \kappa_{\text{RyR}}^3}. \tag{43}$$

Furthermore, r is the fraction of RyR channels that are not inactivated and is governed by the equation

$$\frac{dr}{dt} = -\frac{J_{\text{RyR}}}{\beta_{\text{RyR}}} + \frac{\eta_{\text{RyR}}}{p}(1 - r). \tag{44}$$

Passive diffusion fluxes between compartments The passive diffusion fluxes between intracellular compartments are given by

$$J_d^c = \alpha_d^c(c_d - c_c), \tag{45}$$

$$J_{sl}^c = \alpha_{sl}^c(c_{sl} - c_c), \tag{46}$$

$$J_n^s = \alpha_n^s(c_n - c_s). \tag{47}$$

Buffer fluxes The fluxes of free Ca^{2+} binding to a Ca^{2+} buffer are given by

$$J_d^b = V_d(k_{\text{on}}^d c_d(B_{\text{tot}}^d - b_d) - k_{\text{off}}^d b_d), \tag{48}$$

$$J_{sl}^b = V_{sl}(k_{\text{on}}^{sl} c_{sl}(B_{\text{tot}}^{sl} - b_{sl}) - k_{\text{off}}^{sl} b_{sl}), \tag{49}$$

$$J_c^b = V_c(k_{\text{on}}^c c_c(B_{\text{tot}}^c - b_c) - k_{\text{off}}^c b_c), \tag{50}$$

$$J_s^b = V_s(k_{\text{on}}^s c_s(B_{\text{tot}}^s - b_s) - k_{\text{off}}^s b_s). \tag{51}$$

Membrane fluxes The membrane Ca^{2+} fluxes, J_{CaL}, J_{bCa}, J_{pCa}, and J_{NaCa}, are given by

$$J_{\text{CaL}} = -\frac{\chi C_m}{2F} I_{\text{CaL}}, \qquad J_{\text{pCa}} = -\frac{\chi C_m}{2F} I_{\text{pCa}}, \tag{52}$$

$$J_{\text{bCa}} = -\frac{\chi C_m}{2F} I_{\text{bCa}}, \qquad J_{\text{NaCa}} = \frac{\chi C_m}{F} I_{\text{NaCa}}, \tag{53}$$

where I_{CaL}, I_{bCa}, I_{pCa}, and I_{NaCa} are defined by the expressions given above. Furthermore,

$$J_e^{sl} = J_{\text{NaCa}} + J_{\text{pCa}} + J_{\text{bCa}}. \tag{54}$$

S1.2.3 Na^+ dynamics

The Na^+ concentration is modeled as in [26]

$$\frac{d[\text{Na}_i]}{dt} = -\frac{\chi C_m}{F} \left(I_{\text{Na}} + I_{\text{NaL}} + I_{\text{bNa}} + 3I_{\text{NaK}} + 3I_{\text{NaCa}} + 0.3293 I_{\text{f}} \right), \tag{55}$$

where the currents I_{Na}, I_{NaL}, I_{bNa}, I_{NaK}, I_{NaCa}, and I_{f} are specified above.

S1.2.4 Nernst equilibrium potentials

The Nernst equilibrium potentials for the ion channels are defined as

$$E_{\text{Na}} = \frac{RT}{F} \log\left(\frac{[\text{Na}^+]_{\text{e}}}{[\text{Na}^+]_{\text{i}}}\right), \tag{56}$$

$$E_{\text{Ca}} = \frac{RT}{2F} \log\left(\frac{[\text{Ca}^{2+}]_{\text{e}}}{c_{sl}}\right), \tag{57}$$

$$E_{\text{K}} = \frac{RT}{F} \log\left(\frac{[\text{K}^+]_{\text{e}}}{[\text{K}^+]_{\text{i}}}\right), \tag{58}$$

$$E_{\text{Ks}} = \frac{RT}{F} \log\left(\frac{[\text{K}^+]_{\text{e}} + 0.018[\text{Na}^+]_{\text{e}}}{[\text{K}^+]_{\text{i}} + 0.018[\text{Na}^+]_{\text{i}}}\right), \tag{59}$$

$$E_{\text{Cl}} = \frac{RT}{F} \log\left(\frac{[\text{Cl}^+]_{\text{e}}}{[\text{Cl}^+]_{\text{i}}}\right), \tag{60}$$

$$E_{\text{f}} = -17 \text{ mV}, \tag{61}$$

for the parameter values given in Table S3.

Parameter	Description	Value
V_d	Volume fraction of the dyadic subspace	0.001
V_{sl}	Volume fraction of the SL compartment	0.028
V_c	Volume fraction of the bulk cytosol	0.917
V_s	Volume fraction of the jSR	0.004
V_n	Volume fraction of the nSR	0.05
χ	Cell surface to volume ratio	0.6 μm^{-1}

Table S2: Default geometry parameters of the base model.

Parameter	Description	Value
C_m	Specific membrane capacitance	0.01 pF/μm^2
F	Faraday's constant	96.485 C/mmol
R	Universal gas constant	8.314 J/(mol·K)
T	Temperature	310 K
$[Ca^{2+}]_e$	Extracellular Ca^{2+} concentration	1.8 mM
$[Na^+]_e$	Extracellular sodium concentration	140 mM
$[K^+]_e$	Extracellular potassium concentration	5.4 mM
$[K^+]_e^b$	Baseline extracellular potassium concentration	5.4 mM
$[K^+]_i$	Intracellular potassium concentration	120 mM
$[Cl^-]_e$	Extracellular chloride concentration	150 mM
$[Cl^-]_i$	Intracellular chloride concentration	15 mM

Table S3: Physical constants and ionic concentrations of the base model.

Parameter	Value	Reference
Q_{10}^{Na}	2.0	[35, 28]
Q_{10}^{NaL}	2.2	[35, 28]
Q_{10}^{to}	2.0	[35]
$Q_{10}^{\mathrm{Kr,act}}$	4.55	[35, 36]
$Q_{10}^{\mathrm{Kr,inact}}$	3.08	[35, 36]
Q_{10}^{Ks}	2.0	[35]
Q_{10}^{Kur}	2.2	[37]
Q_{10}^{f}	4.5	[35]
Q_{10}^{CaL}	1.8	[38]
Q_{10}^{NaCa}	1.6	[38]
Q_{10}^{pCa}	2.35	[38]
Q_{10}^{NaK}	1.6	[38]
Q_{10}^{KNaK}	1.5	[38]
Q_{10}^{SERCA}	2.6	[38]
Q^{p}	$\frac{T-310\ \mathrm{K}}{10\ \mathrm{K}}$	

Table S4: Q_{10} values for the base model.

Parameter	Value	Parameter	Value
g_{Na}	10.08 mS/μF	$\bar{I}_{\text{NaK}}$	2.04 μA/μF
g_{NaL}	0.005 mS/μF	$\bar{I}_{\text{NaCa}}$	3.43 μA/μF
g_{to}	0.54 mS/μF	$\bar{I}_{\text{pCa}}$	0.34 μA/μF
g_{Kr}	0.075 mS/μF	$\bar{J}_{\text{SERCA}}$	0.00024 mM/ms
g_{Ks}	0.035 mS/μF	α_{RyR}	0.0045 ms^{-1}
g_{K1}	1.1 mS/μF	β_{RyR}	0.0228 mM
g_{f}	0.0001 mS/μF	α_d^c	0.0017 ms^{-1}
g_{bCl}	0.0082 mS/μF	α_{sl}^c	0.15 ms^{-1}
g_{CaL}	0.389 nL/(μF ms)	α_n^s	0.012 ms^{-1}
g_{bCa}	0.000462 mS/μF	B_{tot}^c	0.07 mM
g_{bNa}	0.0022 mS/μF	B_{tot}^d	1.2 mM
g_{Kur}	1.1 mS/μF	B_{tot}^{sl}	0.9 mM
g_{KACh}	0.0045 mS/μF	B_{tot}^s	27 mM

Table S5: Conductances and similar cell-specific parameter values in the base model formulation. Note that the parameter values of this table defines the canine left atrial version of the base model. For the canine pulmonary vein version, the adjustment factors of Table S6 are applied.

Parameter	PV scaling factor
g_{K1}	0.58
g_{Kr}	1.5
g_{Ks}	1.6
g_{to}	0.75
g_{CaL}	0.7

Table S6: Adjustment factors for the pulmonary vein version of the model, taken from [13].

Parameter	Flux	Value
K_c	J_c^n	0.00025 mM
K_n	J_c^n	1.7 mM
γ_{RyR}	J_s^{sl}	0.001
κ_{RyR}	J_{RyR}	0.015 mM
η_{RyR}	J_s^{sl}	0.00001 ms^{-1}

Table S7: Parameters for the intracellular Ca^{2+} fluxes of the base model.

Parameter	Current	Value
k_{sat}	I_{NaCa}	0.3
ν	I_{NaCa}	0.3
K_{act}	I_{NaCa}	0.00015 mM
$K_{\text{Ca},i}$	I_{NaCa}	0.0036 mM
$K_{\text{Ca},e}$	I_{NaCa}	1.3 mM
$K_{\text{Na},i}$	I_{NaCa}	12.3 mM
$K_{\text{Na},e}$	I_{NaCa}	87.5 mM
$K_{\text{Na},i}^{\text{NaK}}$	I_{NaK}	$(11\text{ mM})\cdot(Q_{10}^{\text{KNaK}})^{Q^p}$
$K_{\text{K},e}$	I_{NaK}	1.5 mM
K_{pCa}	I_{pCa}	0.0005 mM

Table S8: Additional parameters for the membrane currents of the base model.

Parameter	Compartment	Value
k_{on}^c	Bulk cytosol	40 $\text{ms}^{-1}\text{mM}^{-1}$
k_{off}^c	Bulk cytosol	0.03 ms^{-1}
k_{on}^d	Dyad	100 $\text{ms}^{-1}\text{mM}^{-1}$
k_{off}^d	Dyad	1 ms^{-1}
k_{on}^{sl}	Subsarcolemmal space	100 $\text{ms}^{-1}\text{mM}^{-1}$
k_{off}^{sl}	Subsarcolemmal space	0.15 ms^{-1}
k_{on}^s	Junctional SR	100 $\text{ms}^{-1}\text{mM}^{-1}$
k_{off}^s	Junctional SR	65 ms^{-1}

Table S9: Transition rates for the Ca^{2+} buffers of the base model.

Current	Gate	z_∞	α_z	β_z	τ_z
I_{Na}	m	$\frac{1}{\left(1+e^{(-57-v)/9}\right)^2}$	$0.13e^{-((v+46)/16)^2}$	$0.06e^{-((v-5)/51)^2}$	$\frac{\alpha_m+\beta_m}{(Q_{10}^{\mathrm{Na}})^{Q_p}}$
	j	$\frac{1}{\left(1+e^{(v+72)/7}\right)^2}$	0, if $v \geq -40$; $\frac{\begin{pmatrix}-2.5\cdot 10^4 e^{0.2v} \\ -7\cdot 10^{-6} e^{-0.04v}\end{pmatrix}(v+38)}{1+e^{0.3(v+79)}}$, otherwise	$\frac{0.6e^{0.06v}}{1+e^{-0.1(v+32)}}$, if $v \geq -40$; $\frac{0.02e^{-0.01v}}{1+e^{-0.14(v+40)}}$, otherwise	$\frac{1}{(\alpha_j+\beta_j)(Q_{10}^{\mathrm{Na}})^{Q_p}}$
I_{NaL}	m_L	$\frac{1}{1+e^{(-43-v)/5}}$	$\frac{1}{6.8e^{(v+12)/35}}$	$8.6e^{-(v+77)/6}$	$\frac{\alpha_m+\beta_m}{(Q_{10}^{\mathrm{NaL}})^{Q_p}}$
	h_L	$\frac{1}{1+e^{(v+88)/7.5}}$			$\frac{200\text{ ms}}{(Q_{10}^{\mathrm{NaL}})^{Q_p}}$
I_{CaL}	d	$\frac{1}{1+e^{-(v+5)/6}}$	$\frac{1-e^{-\frac{v+5}{6}}}{0.035(v+5)}$		$\alpha_d d_\infty$
	f	$\frac{1}{1+e^{(v+35)/9}}+\frac{0.6}{1+e^{(50-v)/20}}$	$\frac{1}{0.02e^{-(0.034(v+14.5)^2)}+0.02}$		α_f
	f_{Ca}	$\frac{1.7c_d^{1.5}}{1.7c_d^{1.5}+0.012}$	$\frac{1}{1.7c_d^{1.5}+0.012}$		α_{Ca}
I_{to}	q_{to}	$\frac{1}{1+e^{(v+53)/13}}$	$\frac{39}{0.57e^{-0.08(v+44)}+0.065e^{0.1(v+46)}}$	6	$\frac{\alpha_{q_{\mathrm{to}}}+\beta_{q_{\mathrm{to}}}}{(Q_{10}^{\mathrm{to}})^{Q_p}}$
	r_{to}	$\frac{1}{1+e^{-(v-22.3)/18.75}}$	$\frac{14.4}{e^{0.09(v+30.61)}+0.37e^{-0.12(v+24)}}$	2.75	$\frac{\alpha_{r_{\mathrm{to}}}+\beta_{r_{\mathrm{to}}}}{(Q_{10}^{\mathrm{to}})^{Q_p}}$
I_{Kr}	x_{Kr1}	$\frac{1}{1+e^{-(v-2.7)/15.3}}$	$\frac{450}{1+e^{-(v+45)/10}}$	$\frac{6}{1+e^{(v+30)/11.5}}$	$\frac{\alpha_{x_{\mathrm{Kr1}}}\cdot\beta_{x_{\mathrm{Kr1}}}}{(Q_{10}^{\mathrm{Kr,act}})^{Q_p}}$
	x_{Kr2}	$\frac{1}{1+e^{(v+70)/20.9}}$	$\frac{3}{1+e^{-(v+60)/20}}$	$\frac{1.12}{1+e^{(v-60)/20}}$	$\frac{\alpha_{x_{\mathrm{Kr2}}}\cdot\beta_{x_{\mathrm{Kr2}}}}{(Q_{10}^{\mathrm{Kr,inact}})^{Q_p}}$
I_{Ks}	x_{Ks}	$\frac{1}{1+e^{-(v+3.8)/14}}$	$\frac{990}{1+e^{-(v+2.4)/14}}$		$\frac{\alpha_{x_{\mathrm{Ks}}}}{(Q_{10}^{\mathrm{Ks}})^{Q_p}}$
I_{Kur}	x_{Kur1}	$\frac{1}{1+e^{-(v+30.3)/9.6}}$	$\frac{0.65}{e^{-(v+10)/8.5}+e^{-(v-30)/59}}$	$\frac{0.65}{2.5+e^{(v+82)/17}}$	$\frac{1}{(\alpha_{x_{\mathrm{Kr1}}}+\beta_{x_{\mathrm{Kr1}}})(Q_{10}^{\mathrm{Kur}})^{Q_p}}$
	x_{Kur2}	$\frac{1}{1+e^{(v-99.45)/27.48}}$	$\frac{1}{21+e^{-(v-185)/28}}$	$e^{(v-158)/16}$	$\frac{1}{(\alpha_{x_{\mathrm{Kr2}}}+\beta_{x_{\mathrm{Kr2}}})(Q_{10}^{\mathrm{Kur}})^{Q_p}}$
I_{KACh}	x_{KACh}	$\frac{1}{1+e^{(v+93)/15.2}}$	$130\left(1-e^{-(v+130)/50}\right)$	360	$\alpha_{x_{\mathrm{KACh}}}+\beta_{x_{\mathrm{KACh}}}$
I_{f}	x_{f}	$\frac{1}{1+e^{(v+78)/5}}$	$\frac{1900}{1+e^{(v+15)/10}}$		$\frac{\alpha_{x_{\mathrm{f}}}}{(Q_{10}^{\mathrm{f}})^{Q_p}}$

Table S10: Specification of the parameters z_∞ and τ_z, for $z = m$, j, m_L, h_L, d, f, f_{Ca}, q_{to}, r_{to}, x_{Kr1}, x_{Kr2}, x_{Ks}, x_{Kur1}, x_{Kur2}, x_{KACh} and x_{f} in the equations for the gating variables (6).

References

[1] Karoline Horgmo Jæger and Aslak Tveito. Derivation of a cell-based mathematical model of excitable cells. In *Modeling Excitable Tissue*, pages 1–13. Springer, Cham, 2020.

[2] Ruth E Westenbroek, Sebastian Bischoff, Ying Fu, Sebastian KG Maier, William A Catterall, and Todd Scheuer. Localization of sodium channel subtypes in mouse ventricular myocytes using quantitative immunocytochemistry. *Journal of Molecular and Cellular Cardiology*, 64:69–78, 2013.

[3] Esperanza Agullo-Pascual, Xianming Lin, Alejandra Leo-Macias, Mingliang Zhang, Feng-Xia Liang, Zhen Li, Anna Pfenniger, Indra Lübkemeier, Sarah Keegan, David Fenyö, Klaus Willecke, Eli Rothenberg, and Mario Delmar. Super-resolution imaging reveals that loss of the C-terminus of connexin43 limits microtubule plus-end capture and Na_V1. 5 localization at the intercalated disc. *Cardiovascular Research*, 104(2):371–381, 2014.

[4] Rengasayee Veeraraghavan and Steven Poelzing. Mechanisms underlying increased right ventricular conduction sensitivity to flecainide challenge. *Cardiovascular research*, 77(4):749–756, 2007.

[5] Rengasayee Veeraraghavan, Robert G Gourdie, and Steven Poelzing. Mechanisms of cardiac conduction: a history of revisions. *American Journal of Physiology-Heart and Circulatory Physiology*, 306(5):H619–H627, 2014.

[6] Rengasayee Veeraraghavan and Robert G Gourdie. Stochastic optical reconstruction microscopy–based relative localization analysis (STORM-RLA) for quantitative nanoscale assessment of spatial protein organization. *Molecular Biology of the Cell*, 27(22):3583–3590, 2016.

[7] Anthony Varghese. Reciprocal modulation of I_{K1}–I_{Na} extends excitability in cardiac ventricular cells. *Frontiers in Physiology*, 7:542, 2016.

[8] Xianming Lin, Nian Liu, Jia Lu, Jie Zhang, Justus MB Anumonwo, Lori L Isom, Glenn I Fishman, and Mario Delmar. Subcellular heterogeneity of sodium current properties in adult cardiac ventricular myocytes. *Heart Rhythm*, 8(12):1923–1930, 2011.

[9] Diana Shy, Ludovic Gillet, and Hugues Abriel. Cardiac sodium channel $\mathrm{Na_V}1.5$ distribution in myocytes via interacting proteins: the multiple pool model. *Biochimica Et Biophysica Acta (BBA)-Molecular Cell Research*, 1833(4):886–894, 2013.

[10] Alejandra Leo-Macias, Esperanza Agullo-Pascual, Jose L Sanchez-Alonso, Sarah Keegan, Xianming Lin, Tatiana Arcos, Yuri E Korchev, Julia Gorelik, David Fenyö, Eli Rothenberg, and Mario Delmar. Nanoscale visualization of functional adhesion/excitability nodes at the intercalated disc. *Nature Communications*, 7(1):1–12, 2016.

[11] Echrak Hichri, Hugues Abriel, and Jan P Kucera. Distribution of cardiac sodium channels in clusters potentiates ephaptic interactions in the intercalated disc. *The Journal of Physiology*, 596(4):563–589, 2018.

[12] Karoline Horgmo Jæger, Andrew G Edwards, Andrew McCulloch, and Aslak Tveito. Properties of cardiac conduction in a cell-based computational model. *PLoS Computational Biology*, 15(5):e1007042, 2019.

[13] Joachim R Ehrlich, Tae-Joon Cha, Liming Zhang, Denis Chartier, Peter Melnyk, Stefan H Hohnloser, and Stanley Nattel. Cellular electrophysiology of canine pulmonary vein cardiomyocytes: action potential and ionic current properties. *The Journal of Physiology*, 551(3):801–813, 2003.

[14] Nele Vandersickel, Ivan V Kazbanov, Anita Nuitermans, Louis D Weise, Rahul Pandit, and Alexander V Panfilov. A study of early afterdepolarizations in a model for human ventricular tissue. *PloS One*, 9(1):e84595, 2014.

[15] Kirsten HWJ Ten Tusscher and Alexander V Panfilov. Reentry in heterogeneous cardiac tissue described by the Luo-Rudy ventricular action potential model. *American Journal of Physiology-Heart and Circulatory Physiology*, 284(2):H542–H548, 2003.

[16] Karoline Horgmo Jæger, Kristian Gregorius Hustad, Xing Cai, and Aslak Tveito. Efficient numerical solution of the EMI model representing the extracellular space (E), cell membrane (M) and intracellular space (I) of a collection of cardiac cells. *Frontiers in Physics*, 8:539, 2021.

[17] Karoline Horgmo Jæger, Kristian Gregorius Hustad, Xing Cai, and Aslak Tveito. Operator splitting and finite difference schemes for solving the EMI model. In *Modeling Excitable Tissue*, pages 44–55. Springer, Cham, 2020.

[18] Robert Anderson, Julian Andrej, Andrew Barker, Jamie Bramwell, Jean-Sylvian Camier, Jakub Cerveny, Veselin Dobrev, Yohann Dudouit, Aaron Fisher, Tzanio Kolev, Will Pazner, Mark Stowell, Vladimir Tomov, Ido Akkerman, Johann Dahm, David Medina, and Stefano Zampini. MFEM: A modular finite element library. *Computers & Mathematics with Applications*, 2020.

[19] MFEM: Modular finite element methods [Software]. `mfem.org`.

[20] Stanley Rush and Hugh Larsen. A practical algorithm for solving dynamic membrane equations. *IEEE Transactions on Biomedical Engineering*, 4:389–392, 1978.

[21] Joakim Sundnes, Robert Artebrant, Ola Skavhaug, and Aslak Tveito. A second-order algorithm for solving dynamic cell membrane equations. *IEEE Transactions on Biomedical Engineering*, 56(10):2546–2548, 2009.

[22] Johan Hake, Henrik Finsberg, Kristian Gregorius Hustad, and George Bahij. Gotran – General ODE TRANslator, 2020. `https://github.com/ComputationalPhysiology/gotran`.

[23] Tom Lyche. *Numerical Linear Algebra and Matrix Factorizations*, volume 22. Springer Nature, 2020.

[24] Leonardo Dagum and Ramesh Menon. OpenMP: An industry-standard API for shared-memory programming. *IEEE Computational Science and Engineering*, 5(1):46–55, 1998.

[25] Karoline Horgmo Jæger, Verena Charwat, Bérénice Charrez, Henrik Finsberg, Mary M Maleckar, Sam Wall, Kevin Healy, and Aslak Tveito. Improved computational identification of drug response using optical measurements of human stem cell derived cardiomyocytes in microphysiological systems. *Frontiers in Pharmacology*, 10:1648, 2020.

[26] Karoline Horgmo Jæger, Samuel Wall, and Aslak Tveito. Computational prediction of drug response in short QT syndrome type 1 based on measurements of compound effect in stem cell-derived cardiomyocytes. *PLoS Computational Biology*, 17(2):e1008089, 2021.

[27] Eleonora Grandi, Francesco S Pasqualini, and Donald M Bers. A novel computational model of the human ventricular action potential and Ca transient. *Journal of Molecular and Cellular Cardiology*, 48(1):112–121, 2010.

[28] Thomas O'Hara, László Virág, András Varró, and Yoram Rudy. Simulation of the undiseased human cardiac ventricular action potential: Model formulation and experimental validation. *PLoS Computational Biology*, 7(5):e1002061, 2011.

[29] Michelangelo Paci, Jari Hyttinen, Katriina Aalto-Setälä, and Stefano Severi. Computational models of ventricular-and atrial-like human induced pluripotent stem cell derived cardiomyocytes. *Annals of Biomedical Engineering*, 41(11):2334–2348, 2013.

[30] Makarand Deo, Yanfei Ruan, Sandeep V Pandit, Kushal Shah, Omer Berenfeld, Andrew Blaufox, Marina Cerrone, Sami F Noujaim, Marco Denegri, José Jalife, and Silvia G Priori. KCNJ2 mutation in short QT syndrome 3 results in atrial fibrillation and ventricular proarrhythmia. *Proceedings of the National Academy of Sciences*, 110(11):4291–4296, 2013.

[31] Eleonora Grandi, Sandeep V Pandit, Niels Voigt, Antony J Workman, Dobromir Dobrev, José Jalife, and Donald M Bers. Human atrial action potential and Ca^{2+} model: sinus rhythm and chronic atrial fibrillation. *Circulation Research*, 109(9):1055–1066, 2011.

[32] Marc Courtemanche, Rafael J Ramirez, and Stanley Nattel. Ionic mechanisms underlying human atrial action potential properties: insights from a mathematical model. *American Journal of Physiology-Heart and Circulatory Physiology*, 275(1):H301–H321, 1998.

[33] Ingrid E Christophersen, Morten S Olesen, Bo Liang, Martin N Andersen, Anders P Larsen, Jonas B Nielsen, Stig Haunsø, Søren-Peter Olesen, Arnljot Tveit, Jesper H Svendsen, and Nicole Schmitt. Genetic variation in KCNA5: impact on the atrial-specific potassium current I_{Kur} in patients with lone atrial fibrillation. *European heart journal*, 34(20):1517–1525, 2013.

[34] Marta Varela, Michael A Colman, Jules C Hancox, and Oleg V Aslanidi. Atrial heterogeneity generates re-entrant substrate during atrial fibrillation and anti-arrhythmic drug action: mechanistic insights from canine atrial models. *PLoS Computational Biology*, 12(12):e1005245, 2016.

[35] Michelangelo Paci, Elisa Passini, Aleksandra Klimas, Stefano Severi, Jari Hyttinen, Blanca Rodriguez, and Emilia Entcheva. *In silico* populations optimized on optogenetic recordings predict drug effects in human

induced pluripotent stem cell-derived cardiomyocytes. In *2018 Computing in Cardiology Conference (CinC)*, volume 45, pages 1–4. IEEE, 2018.

[36] Maike Mauerhöfer and Christiane K Bauer. Effects of temperature on heteromeric Kv11. 1a/1b and Kv11. 3 channels. *Biophysical Journal*, 111(3):504–523, 2016.

[37] Lasse Skibsbye, Thomas Jespersen, Torsten Christ, Mary M Maleckar, Jonas van den Brink, Pasi Tavi, and Jussi T Koivumäki. Refractoriness in human atria: time and voltage dependence of sodium channel availability. *Journal of Molecular and Cellular Cardiology*, 101:26–34, 2016.

[38] Thomas R Shannon, Fei Wang, José Puglisi, Christopher Weber, and Donald M Bers. A mathematical treatment of integrated Ca dynamics within the ventricular myocyte. *Biophysical Journal*, 87(5):3351–3371, 2004.
